# Anterior Overgrowth in Primary Curves, Compensatory Curves and Junctional Segments in Adolescent Idiopathic Scoliosis

**DOI:** 10.1371/journal.pone.0160267

**Published:** 2016-07-28

**Authors:** Tom P. C. Schlösser, Marijn van Stralen, Winnie C. W. Chu, Tsz-Ping Lam, Bobby K. W. Ng, Koen L. Vincken, Jack C. Y. Cheng, René M. Castelein

**Affiliations:** 1 Department of Orthopaedic Surgery, University Medical Center Utrecht, Utrecht, The Netherlands; 2 Image Sciences Institute, University Medical Center Utrecht, Utrecht, The Netherlands; 3 Department of Diagnostic Radiology and Organ Imaging, Prince of Wales Hospital, The Chinese University of Hong Kong, Shatin, Hong Kong; 4 Department of Orthopaedics and Traumatology, Prince of Wales Hospital, The Chinese University of Hong Kong, Shatin, Hong Kong; Rush University Medical Center, UNITED STATES

## Abstract

**Introduction:**

Although much attention has been given to the global three-dimensional aspect of adolescent idiopathic scoliosis (AIS), the accurate three-dimensional morphology of the primary and compensatory curves, as well as the intervening junctional segments, in the scoliotic spine has not been described before.

**Methods:**

A unique series of 77 AIS patients with high-resolution CT scans of the spine, acquired for surgical planning purposes, were included and compared to 22 healthy controls. Non-idiopathic curves were excluded. Endplate segmentation and local longitudinal axis in endplate plane enabled semi-automatic geometric analysis of the complete three-dimensional morphology of the spine, taking inter-vertebral rotation, intra-vertebral torsion and coronal and sagittal tilt into account. Intraclass correlation coefficients for interobserver reliability were 0.98–1.00. Coronal deviation, axial rotation and the exact length discrepancies in the reconstructed sagittal plane, as defined per vertebra and disc, were analyzed for each primary and compensatory curve as well as for the junctional segments in-between.

**Results:**

The anterior-posterior difference of spinal length, based on “true” anterior and posterior points on endplates, was +3.8% for thoracic and +9.4% for (thoraco)lumbar curves, while the junctional segments were almost straight. This differed significantly from control group thoracic kyphosis (-4.1%; *P*<0.001) and lumbar lordosis (+7.8%; *P*<0.001). For all primary as well as compensatory curves, we observed linear correlations between the coronal Cobb angle, axial rotation and the anterior-posterior length difference (*r*≥0.729 for thoracic curves; *r*≥0.485 for (thoraco)lumbar curves).

**Conclusions:**

Excess anterior length of the spine in AIS has been described as a generalized growth disturbance, causing relative anterior spinal overgrowth. This study is the first to demonstrate that this anterior overgrowth is not a generalized phenomenon. It is confined to the primary as well as the compensatory curves, the junctional zones do not exhibit this growth discrepancy, however, they are straight.

## Introduction

Anterior overgrowth, or excess anterior length of the spine, is a characteristic of idiopathic scoliosis and has been presented as its possible cause.[1–4] In order to keep the head of the patient over the pelvis, the spine is forced to twist into a curvature, thus providing room for the longer anterior structures without losing postural balance.

The question whether this disturbance of harmonious anterior-posterior growth acts on the scoliotic spine as a whole, or whether it is restricted to the primary curve, may include the compensatory curve(s) and possibly also the intervening junctional zones has never been addressed before and forms the purpose of this study. The aim of this study was to accurately define the three-dimensional (3-D) morphology of the different areas in the spine in adolescent idiopathic scoliosis (AIS) patients in terms of anterior-posterior length difference, coronal deformation and axial rotation. We hypothesize that the anterior-posterior length difference in primary AIS curves is similar to the morphology of the compensatory curves, that the anterior-posterior length difference of the scoliotic spine is different from controls and that the complete 3-D deformity is correlated with curve magnitude.

## Materials and Methods

### Study population

UMC Utrecht and Chinese University of Hong Kong Ethical Review Board approved this study. Participants did not provide informed consent, because this was a retrospective study on pre-existing hospital records and clinical course was not modified. All patients, meeting criteria for AIS surgery and agreeing to surgery (age at diagnosis between 10 and 18), who had undergone CT scanning for CT guided pedicle screw placement and complete preoperative workup (standing posterior-anterior, lateral and supine bending radiographs as well as full-spine MRI, obtained for exclusion of neural axis abnormalities) in an academic center in Hong Kong between July 2009 and February 2014, were included. Patients with non-idiopathic scoliosis (including congenital malformations, syringomyelia and Chiari malformation), left-convex thoracic curves or any other pathology of the spine, previous spinal surgery, syndromes associated with muscle weakness, growth abnormalities or incomplete preoperative workup were excluded. Clinical and radiological charts were reviewed of all patients for in- and exclusion. CT scans (T4-S1, voxel dimensions 0.6*0.3*0.3mm, 64 Slice Multi-detector CT scanner, GE Healthcare, Chalfont, United Kingdom) were part of the pre-operative protocol and had been obtained in prone position, as in the the positioning during surgery. Using the radiographs, conventional parameters were measured (Cobb angles, thoracic kyphosis (T4–T12), lumbar lordosis (L1–S1) and Cobb angle correction on the bending radiographs[5]) and curves were classified according to Lenke’s criteria[6], into primary and compensatory, main thoracic and (thoraco)lumbar curves.

Twenty-two age- and sex matched healthy adolescents without spinal pathology were selected from a database of patients who had undergone full-body CT examination in supine position for trauma screening as a reference for normal anatomy.

### CT measurement method

**Fig 1:**
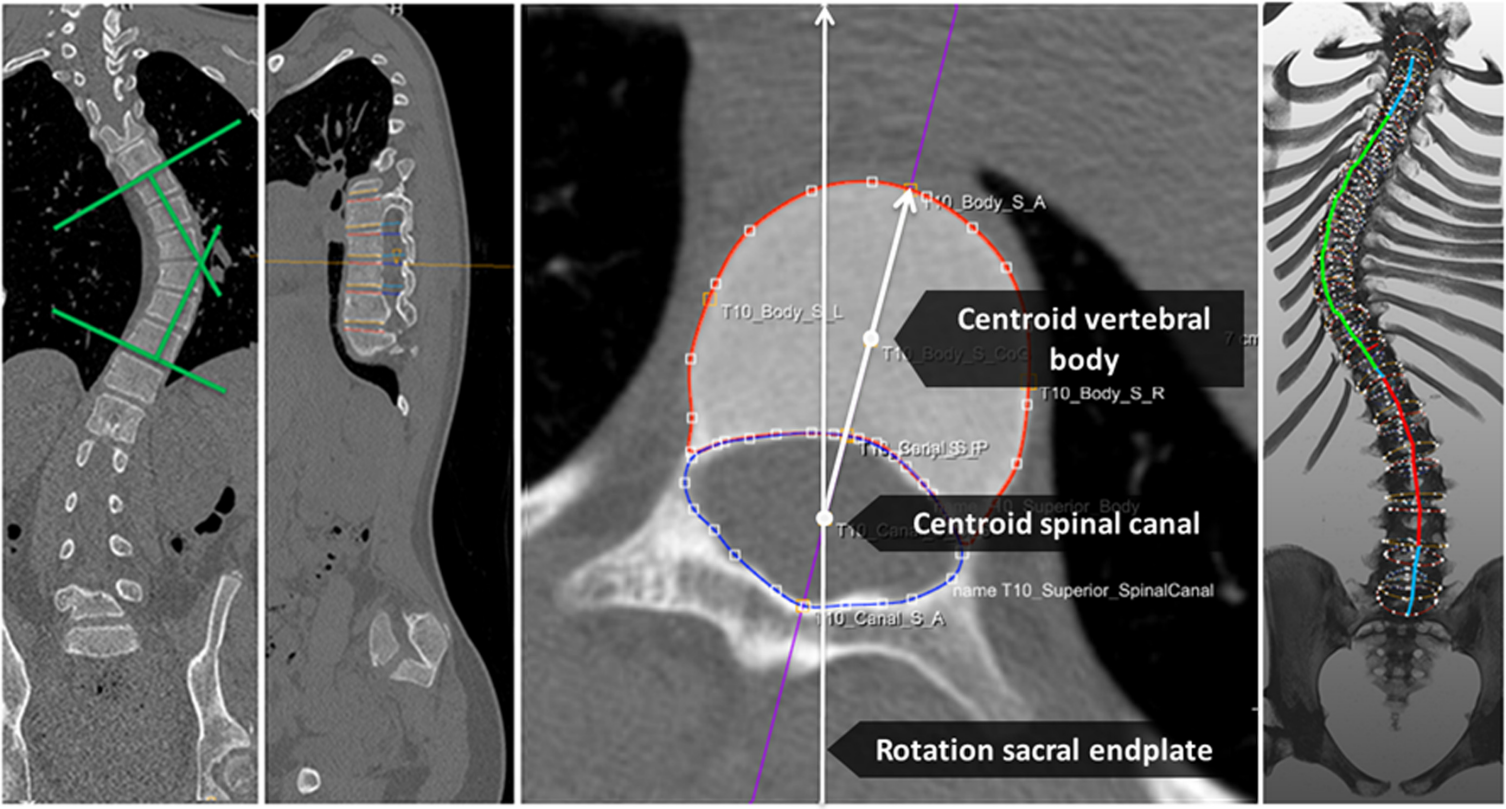
Cobb angle measurements for coronal deviation of the main thoracic and (thoraco)lumbar curve was performed on the coronal slice on which the curve was best visible. Anterior-posterior axis of the endplate was automatically found as the line through the centroid of the spinal canal segmentation and centroid of the endplate segmentation. Based on the anterior-posterior axis, the coordinates of the most anterior and most posterior part of each endplate was found. Length was calculated as a summation of distances between these points for the anterior and posterior aspects of the endplate, and for each vertebra and for each disc, respectively. The total anterior length of the main thoracic curve and (thoraco)lumbar curve are illustrated in *green* and *red* on a 3-D spinal reconstruction, respectively.

Semi-automatic analysis software (ScoliosisAnalysis, Image Sciences Institute, Utrecht, the Netherlands) was developed using MeVisLab (MeVis Medical Solutions AG, Bremen, Germany) to measure the coronal Cobb angle, axial rotation and corrected anterior-posterior length discrepancy of the different spinal areas, taking inter-vertebral rotation, intra-vertebral torsion and coronal and sagittal tilt of each individual endplate into account on the CT scan. This was assessed for the primary and compensatory main thoracic and (thoraco)lumbar curves as well as the proximal and distal junctional segments and compared to the healthy controls (Fig 1). Since the control cohort did not have scoliosis, we used representative levels for thoracic and lumbar anatomy: T4 to T12 and L1 to L5.

For the measurement of axial rotation and the anterior-posterior length discrepancy, first the orientation of all upper and lower endplates between spinal levels T1 and the sacral plate was determined double oblique, by manual delineation on the coronal and sagittal plane. This enabled automatic reconstruction of the ‘true’ transverse section of each individual upper and lower endplate. Second, on these sections, the endplate and spinal canal were segmented manually and the longitudinal axis of each individual endplate was automatically found based on the centroids of the spinal canal and vertebral body segmentations, using a technique previously validated by Kouwenhoven *et al*.. [7] Third, based on the segmentations and longitudinal axis, the coordinates of the exact anterior and posterior side of each superior and inferior endplate were determined automatically. Using the distance between the anterior coordinate of the superior and inferior endplates in the world coordinate system, the ‘true’ anterior height of the vertebrae and discs in-between the vertebrae were calculated. In the same way, posterior height of the vertebrae and discs were calculated. Length was calculated as a summation of distances between these points for the anterior and posterior aspects of the endplate, and for each vertebra and for each disc, respectively. By this technique, curve length measurements were performed at the level of the anterior and posterior aspect of the vertebral bodies and discs and the posterior elements were not included in the analyses.

Based on the complete 3-D reconstructions and multiple oblique sagittal reconstructions, the true sagittal length of the anterior and posterior side of the curves was calculated from Cobb end vertebra to Cobb end vertebra, and the apical and junctional segments, taking 3-D lateral deviation, rotation and tilt of each vertebra into account. The distal junctional segment was represented by the lower thoracic end vertebra, one vertebra below and the disc in-between, and the proximal junctional segment as the upper end vertebra, one level above and the disc in-between. The apical region was defined as the apical vertebra and the two adjacent vertebrae and discs. To be able to compare the anterior-posterior length discrepancy between all subjects and spinal regions, relative anterior-posterior length differences (Δ(A-P)) were calculated, Δ(A-P) = (anterior length − posterior length) / posterior length × 100%. Positive values indicated greater anterior curve length, negative values greater posterior length, relative to total posterior length of the curve.

Axial rotation was calculated for each endplate from T4 to L5. This was defined as the angle between each anterior-posterior axis and the anterior-posterior axis of the sacral plate, since S1 is not normally part of the deformity in idiopathic scoliosis, in the transverse plane. Right-sided rotation was assigned a positive value.

Measurements were performed by two trained observers. For assessment of variability between observers, they independently analyzed a subset of 10 randomly selected CT scans at separate sittings. Intraclass correlation coefficients (ICC) for all parameters were calculated to assess the inter-observer variability.

### Statistical analysis

Statistical analyses were performed using SPSS 20.0 (SPSS, Inc., Chicago, IL, United States). Mean and standard deviations (SD) were calculated for all continuous parameters. Kolmogorov-Smirnov tests were used to test for normality of distribution. For comparisons between AIS and controls, and between curve types (primary and compensatory curves), independent samples t-tests and one-way ANOVA were used, respectively. Bonferroni’s correction was applied for *post hoc* analyses. For comparisons between apical and junctional segments, paired samples t-tests were used. At last, we evaluated the relationship between the 3-D parameters in the different planes with conventional parameters on the pre-operative standing radiographs using scatterplots and Pearson’s correlation coefficient (*r*). The sample size was determined for an independent sample t-test to detect differences between primary curves and secondary curves for a power of 80% and type II error 0.05 using preliminary data from previous study (mu1 = 3.8, mu2 = 2.5, sigma = 2.8). The significance level was set at 0.05.

## Results

### Demographics

Eleven out of eighty-eight patients were excluded because they had a congenital scoliosis (n = 4), a syrinx on the pre-operative MRI scan (n = 1), Marfan’s disease (n = 1), undergone previous spinal surgery (n = 1), left thoracic curve (n = 3) or incomplete pre-operative workup (n = 1). Demographics and curve characteristics of the seventy-seven included AIS subjects and twenty-two controls are shown in Table 1. All CT scans of the AIS cases and controls included all levels from T4 to S1, the average radiation dose was 10 mSv.

**Table 1:** Demographic data are shown for the adolescent idiopathic scoliosis (AIS) patients and matched controls.

	AIS (n = 77)	Controls (n = 22)
Mean age in years ± SD	17.0±2.8	17.0±0.7
Girls, n	60 (78%)	16 (73%)
Triradiate cartilage closed	100%)	
Risser’s stage 0	0 (0%)	
Risser’s stage 1	5 (6%)	
Risser’s stage 2	1 (1%)	
Risser’s stage 3	8 (10%)	
Risser’s stage 4	16 (21%)	
Risser’s stage 5	47 (61%)	
***Primary thoracic curve pattern***	53 (69%)	
Lenke I	37 (48%)	
Lenke II	16 (21%)	
***Primary (thoraco)lumbar curve pattern***	6 (8%)	
Lenke V	6 (8%)	
***Double curve pattern***	18 (23%)	
Lenke III (primary thoracic)	7 (9%)	
Lenke IV (primary thoracic)	2 (3%)	
Lenke VI (primary (thoraco)lumbar)	9 (12%)	

SD = standard deviation.

### Anterior overgrowth in AIS as compared to controls

All thoracic AIS curves were significantly longer anterior than posterior, and normalized anterior length was greater in scoliosis (+3.8±2.8%) than in controls (-4.1±1.8%) over the same segments (*P*<0.001; Table 2). Thoracic AIS curves were on average 7.9% longer anteriorly over the same segments than the controls. As a reference, the mean absolute anterior and posterior length of the thoracic AIS curves was 153±33mm and 147±31mm, respectively. The (thoraco)lumbar AIS curves were 9.4±2.5% longer anteriorly, whereas the lumbar spines of controls were 7.8±3.6% longer anteriorly (*P*<0.021); indicating an increase of the normal lumbar lordosis in the AIS subjects.

**Table 2:** Differences in three-dimensional measurements are shown for adolescent idiopathic scoliosis (AIS) subjects and the thoracic (T4-T12) and lumbar (L1-L5) spine in matched controls.

	AIS (n = 77)	Controls (n = 22)	*P*
**Main thoracic curve parameters:**			
*Coronal Cobb (°)*	58 ± 14	n.a.	n.a.
*Axial rotation (°)*	+24 ± 9	+1 ± 3	<0.001
Δ(A-P)* *(%)*	+3.8 ± 2.8	-4.1 ± 1.8	<0.001
**(Thoraco)lumbar curve parameters:**			
*Coronal Cobb (°)*	37 ± 16	n.a.	n.a.
*Axial rotation (°)*	-9 ± 9	-1 ± 5	0.001
*Δ(A-P) (%)*	+9.4 ± 2.5	+7.8 ± 3.6	0.021

Δ(A-P) = anterior-posterior length discrepancy; n.a. = not applicable; n.s. = not significant.

* Positive values indicated greater anterior curve length. Percentages are shown relative to total posterior length of the curve.

### Anterior overgrowth in different spinal regions

In all curves, primary as well as compensatory, the anterior length exceeded the posterior length of the curve from Cobb to Cobb end vertebra (Table 3). Most anterior overgrowth was observed in the apical regions of the thoracic and (thoraco)lumbar curves (Table 4). In contrast, in the proximal and distal junctional segments between the primary and compensatory curves, no clear anterior-posterior length discrepancy was observed (*P*<0.001).

**Table 3:** Differences in three-dimensional measurements are shown for adolescent idiopathic scoliosis subjects with three different curve types.

	Primary Thoracic curve pattern (n = 53)	Primary (Thoraco)lumbar curve pattern (n = 6)	Double curve pattern (n = 18)	*P*
**Thoracic curve parameters:**				
*Primary/compensatory*	*Primary*	*Compensatory*	*Structural*, *primary or compensatory*	
*Coronal Cobb (°)*	60 ± 11	40 ± 13†	61 ± 17	0.001
*Axial rotation (°)*	26 ± 7	14 ± 6†	24 ± 12	0.004
Δ(A-P) (%)*	4.2 ±2.7	1.8 ± 2.2	3.6 ± 3.1	>0.05
(**Thoraco)lumbar curve parameters:**				
*Primary/compensatory*	*Compensatory*	*Primary*	*Structural*, *primary or compensatory*	
*Coronal Cobb (°)*	28 ± 10†	57 ± 12	55 ± 12	<0.001
*Axial rotation (°)*	- 4 ± 5†	- 20 ± 6	- 19 ± 8	<0.001
Δ(A-P) (%)*	8.6 ± 2.2†	10.8 ± 2.3	11.4 ± 2.2	0.001

Δ(A-P) = anterior-posterior length discrepancy

†*P*<0.05 in *post hoc* analysis.

* Positive values indicated greater anterior curve length. Percentages are shown relative to total posterior length of the curve.

**Table 4:** Percentage anterior-posterior length discrepancy and axial rotation are shown for different regions of the scoliotic spine. Δ(A-P) = anterior-posterior length discrepancy.

	**Proximal junctional segment**	**Apical zone thoracic curve**	**Distal junctional segment**	**Apical zone (thoraco)lumbar curve**	***P***
Δ(A-P) (%)*	-1.3 ± 4.7	7.1 ± 4.8	1.0 ± 2.7	11.2 ± 4.1	<0.001
Axial rotation (°)	5 ± 9	24 ± 9	- 1 ± 11	- 9 ± 9	<0.001
	**Proximal junctional segment**	**Apical zone thoracic curve**	**Distal junctional segment**	**Apical zone (thoraco)lumbar curve**	***P***
Δ(A-P) (%)*	-1.3 ± 4.7	7.1 ± 4.8	1.0 ± 2.7	11.2 ± 4.1	<0.001
*Axial rotation (°)*	5 ± 9	24 ± 9	- 1 ± 11	- 9 ± 9	<0.001

* Positive values indicated greater anterior curve length. Percentages are shown relative to total posterior length of the curve.

### Coupling between the three-dimensions

**Fig 2:**
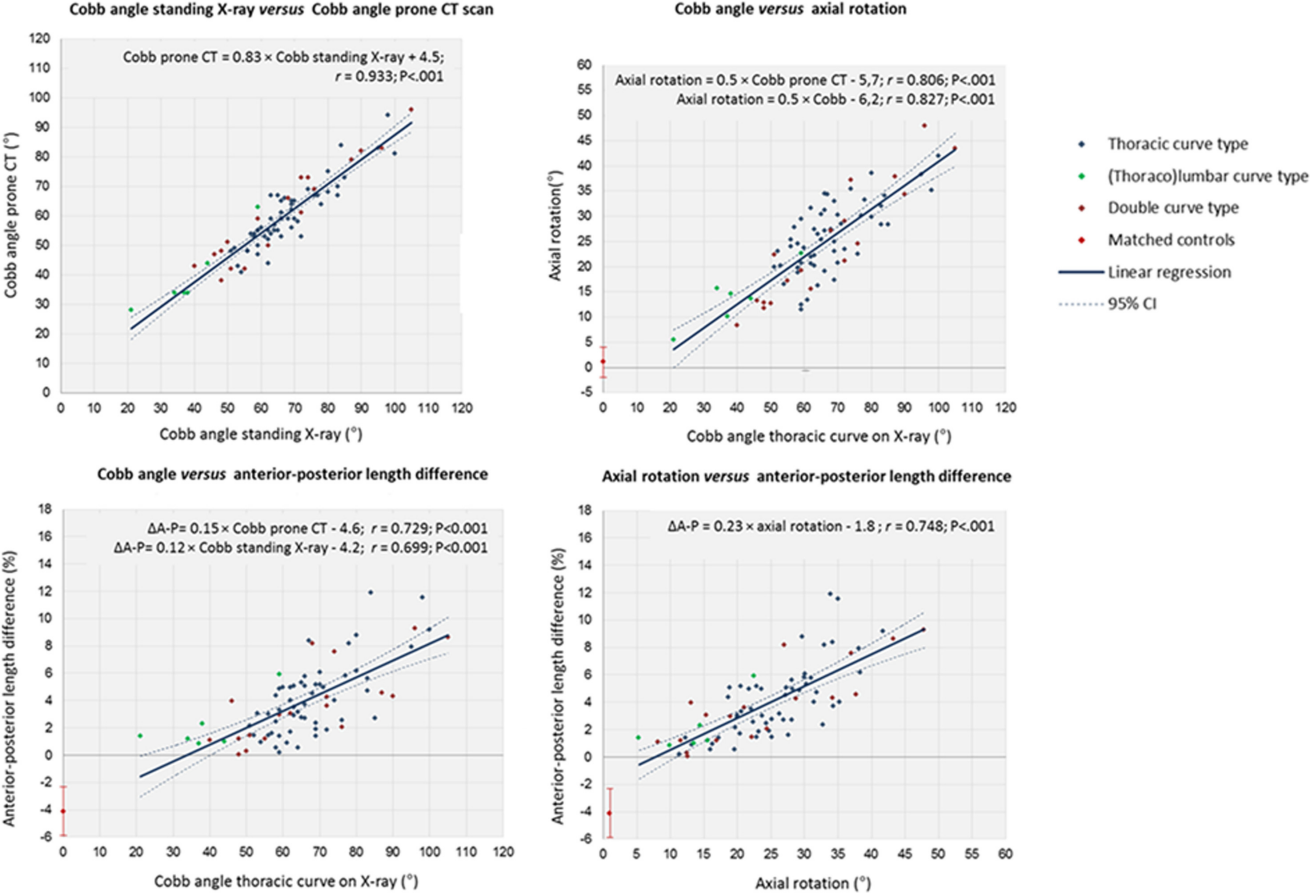
Correlation between Cobb angle of the main thoracic curve on standing radiographs *versus* prone CT scans, between the deformation in three-dimensions. Pearson’s correlation coefficients (*r*), linear regression equations and significance levels are shown including the 95% confidence interval (95% CI). For the control group, mean and standard deviations (error bars) are shown.

Pearson’s correlation analysis revealed a statistically significant correlation between the upright coronal Cobb angle on the standard X-ray, the Cobb angle on the reformatted CT, axial rotation and ΔA-P (*r* = 0.806, *P*<0.001 and *r =* 0.729, *P*<0.001, respectively). Rotation and ΔA-P also correlated significantly with each other (*r* = 0.748, *P*<0.001). Additionally, significant correlations and linear relations were observed between Cobb angle measurements on the standing radiograph and axial rotation and Δ(A-P) on the CT scans (*P*<0.001). For practical purposes, with every 10° increase in Cobb angle of the primary or compensatory thoracic curve on the standing radiograph, axial rotation increased 5° and the relative anterior length of the curve increased 1.2%. Scatterplots and equations are demonstrated in Fig 2.

### Reliability of measurements

ICC’s for inter-observer measurements reliability were 0.98 (95%CI: 0.97–0.99) for Cobb angle, 0.99 (0.99–1.00) for axial rotation and 0.97 (0.92–0.98) for the anterior-posterior length discrepancy calculations. The error of our measurements of anterior and posterior height of the spinal regions was 0.2mm.[8]

## Discussion

Laminae and vertebral bodies ossify in a different manner; normally there is a synchronous coupling of membranous ossification of the posterior elements (the pedicles, laminae and articular, transverse and spinous processes) and the endochondral ossification of the vertebral bodies, that keeps pace with the development of the central nervous system. In AIS this coupling is disturbed, leading to the phenomenon that is called “relative anterior overgrowth”.[1–4] Due to accelerated anterior growth, normal thoracic kyphosis is replaced by a lordosis, that has to rotate sideways around a posterior axis in order to balance the patient’s head over the pelvis.[9–11] The reason for this uncoupling of normal harmonious anterior and posterior growth is thought to lie in a relative tether by the spinal cord, that was shown to have a normal length in the presence of an abnormally long anterior spinal column. This has led to the concept of asynchronous neuro-osseous growth as one of the prevailing etio-pathogenetic mechanisms in the development and progression of idiopathic scoliosis.[1–4]

**Fig 3:**
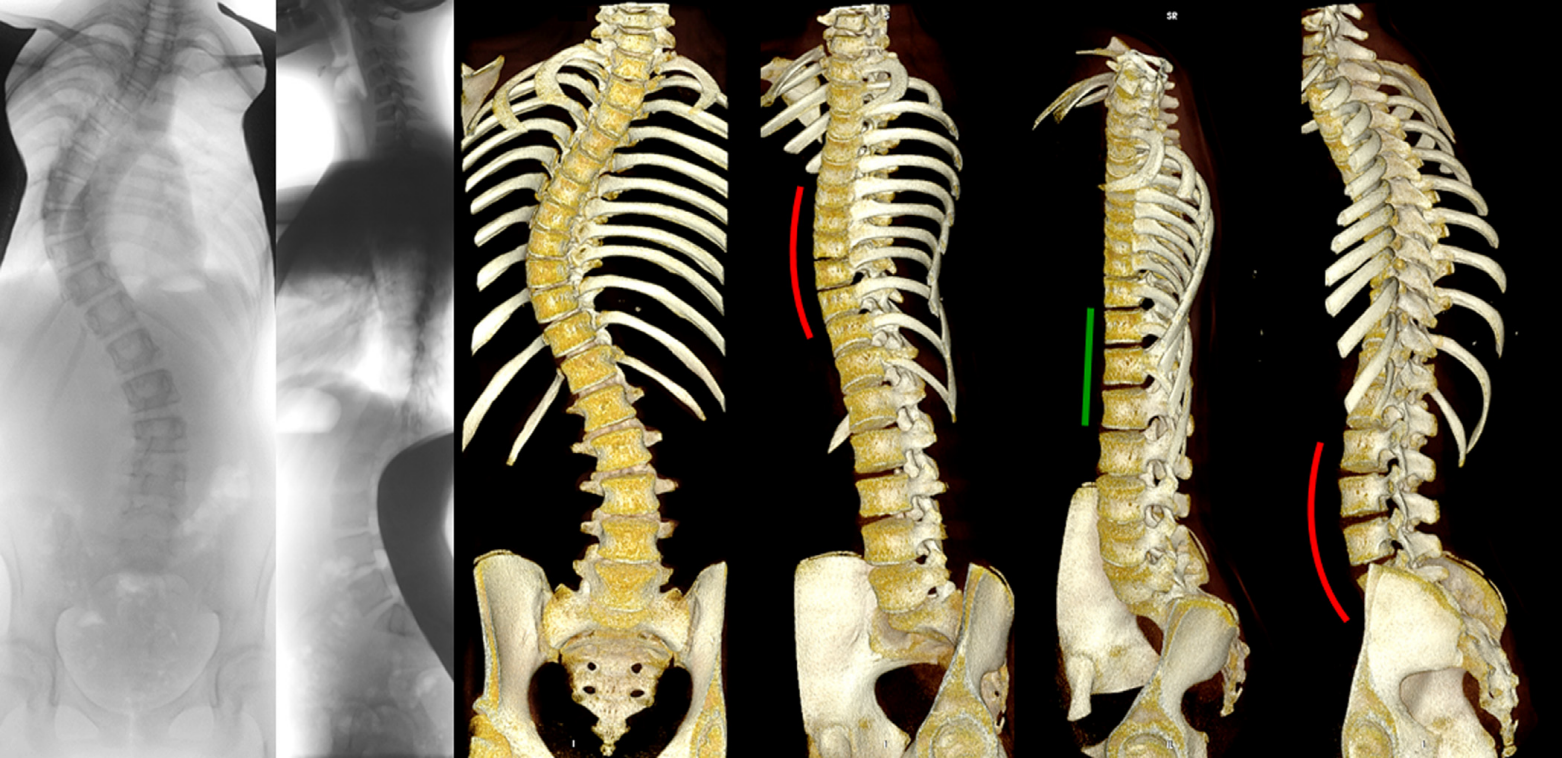
Posterior-anterior and lateral radiographs, and computed tomography reconstructions (OsiriX 64-bit; OsiriX Foundation, Los Angeles, CA, USA) are shown from an anterior and anterolateral view. In this representative 15-year-old patient with adolescent idiopathic scoliosis with a typical thoracic curve pattern, anterior overgrowth cannot be observed on the lateral radiography. The rotated lordoses of both thoracic and the (thoraco)lumbar curvatures, with a clearly longer anterior spinal column than posterior elements, are shown in *red*, and the straight junctional segment in-between in *green* from a true lateral view for these regions.

Different areas with different characteristics can usually be recognized in the scoliotic spine; a primary curve, secondary curve(s) and junctional zones. The primary curve is considered the origin of the deformity. Despite many years of dedicated research into the etio-pathogenesis of AIS, no uniform cause has been established for the development of the primary curve and the disorder is therefore still called multi-factorial.[12–14] The compensatory curve is usually smaller, less rotated and more flexible than the primary, it is considered a consequence of the primary curve in an attempt of the body to restore balance.[6] These curved areas are connected by the junctional zones, that typically are the most tilted but least rotated areas of the spine in scoliosis. Although an overgrowth of the anterior spinal column in idiopathic scoliosis has been demonstrated in a number of studies, our study is, to the best of our knowledge, the first to provide an accurate quantitative description of the regional morphology of the primary and compensatory curves as well as the junctional zones in AIS using high-resolution 3-D scans (Fig 3).

3-D CT reconstructions are considered the ‘gold standard’ for quantification of the morphology of *in vivo* structures.[15] In scoliosis, all vertebrae and, due to intrinsic torsion of vertebrae and discs, all endplates are orientated in a different direction.[8] For this reason we used the orientation of each individual endplate, instead of ‘vertebra-vectors’ as used in biplanar radiography 3-D reconstructions.[16] By taking vertebral rotation and torsion as well as sagittal and coronal tilting of each individual endplate into account, we were able to calculate the complete 3-D profile of the curvatures and junctional zones of the spine, including the ‘true’ anterior and posterior length discrepancy.

This is definitely not the first morphometric investigation in which the corrected sagittal contour of AIS curvatures was studied in detail and compared to normal anatomy, and the apical lordosis in scoliosis was described before.[11,17–22] Porter reported on differences in total axial length of the vertebral bodies and spinal canal in skeletons of scoliotic patients and normals. He observed relatively shorter spinal canals in the scoliotic spines and found a relation of the length discrepancy with the severity of the curves.[1,2] More recently, Chu *et al*. and Guo *et al*. reported shorter spinal canals, decreased interpedicular distance and a relatively longer anterior spinal column on reformatted magnetic resonance images of AIS patients compared to controls.[3,4] Recently, Hayashi *et al*. and Newton *et al*. came to a similar conclusion in a study using spinal reconstructions of biplanar radiographs.[18,23] As far as the authors know, our study, however, is the first to completely correct for the 3-D deformation including intra-vertebral rotation, and none of the previous studies differentiated between the different regions of the spine.

The anterior side of the vertebral bodies and discs in scoliosis is definitely longer than the posterior side of the bodies and discs, but anterior overgrowth is not a generalized phenomenon throughout the spine. It occurs most around the apex of the curve, whether it is the primary or the compensatory, but it does not exist in the junctional zones. Moreover, the changes in the coronal, axial and sagittal plane showed a linear relationship for both curves in double curve deformities: these are coupled phenomena. Due to different rib geometry, curves with similar 3-D characteristics may still exhibit differences in cosmetic appearance.

Our findings add to the knowledge and understanding of the complex 3-D deformity that AIS is. Conclusions on scoliosis causation is beyond the capabilities of this cross-sectional study. In this study the same linear relationship between coronal deformity and anterior-posterior length difference was found on both curves in a double-curve deformity, but not in junctional regions. Because the same association of frontal plane curvature and anterior overgrowth is present on both primary and secondary curves, we speculate that it seems unlikely the anterior overgrowth ‘drives’ the progression of deformity. Moreover, if a primary growth disturbance is the cause of idiopathic scoliosis, it apparently only acts on the apical zones and not on the junctional segments, which should focus future research to the differences between these different areas of the spine. For practical purposes, it highlights the difficulty of surgically restoring thoracic kyphosis in a segment of the spine that is a rotated lordosis to begin with.

The same series of high-resolution CT-scans and software was previously used for a study on the individual contribution of the vertebral bodies as compared to the discs to the 3-D deformity in AIS.[8] It was found that the intervertebral discs were at least three times more deformed in the coronal, true transverse and true sagittal plane than the vertebral bodies. Conclusions from this and the previous study are that excess of anterior length is not a global, but rather a regional phenomenon that is most pronounced in the apical discs in the primary as well as secondary curves.

Drawbacks of our study are the cross-sectional design, that the images were not obtained upright, as is the standard in scoliosis, and that ionizing radiation was used. Although the general configuration of the spine is definitely affected by positioning, we feel that our observations are still valid. If the excess anterior length around the apex of the curve was caused by positioning, the primary and secondary curves as well as the junctional zones would react the same, unless there is a real and structural difference between these distinct areas of the spine. Regarding the use of CT scans, for this study we used an already existing database of images originally acquired for facilitating the initial planning of the navigation guided pedicle screw insertion in posterior instrumentation. Further routine CT scan are not recommended for future surgical treatment or research of AIS patients.

## Conclusions

This study demonstrates a correlation between the deformity in three planes in primary as well as compensatory AIS curves, where greater coronal Cobb angles correlate with greater rotation and more anterior lengthening of the spine. Our data confirm anterior overgrowth in idiopathic scoliosis, and better define the true regional nature: it is not a generalized growth disturbance of the spine but is confined to the area around the apex of both the primary and the secondary curve.

## Supporting Information

S1 Data set (XLSX)
